# Genetic Polymorphisms of *miR-146a* and *miR-27a*, *H. pylori* Infection, and Risk of Gastric Lesions in a Chinese Population

**DOI:** 10.1371/journal.pone.0061250

**Published:** 2013-04-17

**Authors:** Ming-yang Song, Hui-juan Su, Lian Zhang, Jun-ling Ma, Ji-you Li, Kai-feng Pan, Wei-cheng You

**Affiliations:** 1 Key Laboratory of Carcinogenesis and Translational Research (Ministry of Education), Department of Cancer Epidemiology, Peking University Cancer Hospital and Institute, Beijing, People’s Republic of China; 2 Department of Pathology, Peking University Cancer Hospital and Institute, Beijing, People’s Republic of China; Sapporo Medical University, Japan

## Abstract

**Background:**

MicroRNAs (miRNAs) have been implicated in various human diseases. Single nucleotide polymorphisms (SNPs) in inflammation-related miRNA may play an important role in *Helicobacter pylori* (*H. pylori*)-induced gastric lesions. To evaluate the associations between miRNA SNPs, *H. pylori* and gastric lesions, a population-based study was conducted in Linqu County, China.

**Methodology/Principal Findings:**

Based on serum miRNA array conducted in this population, two SNP loci (*miR-146a* rs2910164: G>C and *miR-27a* rs895819: T>C) were determined by polymerase chain reaction-restriction fragment length polymorphism in 2,380 participants with diverse gastric lesions. Using participants with superficial gastritis and mild chronic atrophic gastritis as the reference group, we found that rs2910164 CC carriers had a significantly increased risk of intestinal metaplasia [adjusted odds ratio (OR), 1.42; 95% confidence interval (CI), 1.03–1.97] and dysplasia (OR, 1.54; 95% CI, 1.05–2.25) compared to GG carriers, whereas no significant association was observed for rs895819. Stratified analysis by *H. pylori* infection indicated that rs2910164 C allele was associated with an increased risk of intestinal metaplasia and dysplasia only among individuals infected with *H. pylori* (CC vs. GG: OR, 1.53; 95% CI, 1.12–2.08, *P* for trend = 0.004). Participants who simultaneously carried variant alleles and *H. pylori* infection were more likely to develop intestinal metaplasia and dysplasia, although the interaction between genetic variants and *H. pylori* infection was not significant (*P* for interaction = 0.35 for rs2910164 and 0.92 for rs895819).

**Conclusions/Significance:**

These findings suggest that *miR-146a* rs2910164 polymorphism may contribute to the evolution of *H. pylori*-associated gastric lesions in this high-risk population.

## Introduction

*Helicobacter pylori* (*H. pylori*) is a gram-negative bacterial pathogen and has been recognized as the major cause of gastric cancer (GC) [1]. Chronic gastritis induced by *H. pylori* can persist for decades and may progress to intestinal metaplasia (IM), dysplasia (DYS) and intestinal-type GC [2]. Although about half of the world’s population is infected with *H. pylori*, only a small proportion of infected people ultimately develop GC [3], suggesting that genetic polymorphisms in inflammation-related genes may play a role in this process [4], [5]. Recent studies demonstrate that single nucleotide polymorphisms (SNPs) in microRNAs (miRNAs) could also affect cancer risk [6].

MiRNAs are an abundant class of small non-coding RNAs that involve in various biological processes and human diseases by negatively regulating the translational efficiency and stability of their target mRNAs [7]–[9]. Studies have shown that SNP or mutation in miRNA sequence may influence cancer susceptibility by altering miRNA expression, maturation or miRNA–mRNA interaction [6], [10], [11]. In recent years, we focused on the evaluation of circulating miRNAs in early detection of GC, and identified the differentially expressed miRNAs in GC through genome-wide serum miRNA expression profiling [12]. Based on this array data, we searched for potential functional SNP loci which could be involved in *H. pylori*-induced inflammatory process. Among them, we selected two SNPs for the current study, *miR-146a* rs2910164: G>C and *miR-27a* rs895819: T>C, which have been reported to influence GC risk [13], [14].

*MiR-146a* is involved in the regulation of innate immunity and *H. pylori*-induced inflammatory response through modulating the expression of target genes, IL-1 receptor-associated kinase 1 (*IRAK1*) and TNF receptor-associated factor 6 (*TRAF6*) [15], [16]. *miR-27a* has been shown to function as oncogenes in gastric adenocarcinoma by targeting prohibitin and forkhead box protein O1 (*FOXO1*) [13], [17], which could protect cells against oxidative stress [18], [19]. Because *H. pylori* infection-induced inflammation is an important source of oxidative stress by producing reactive oxygen species (ROS), we also selected *miR-27a* as a candidate. The polymorphisms of rs2910164 in *miR-146a* and rs895819 in *miR-27a* have been reported to affect the corresponding miRNA production [20], 21 and GC susceptibility [14], [22]. However, there are limited data on their relationships with *H. pylori*-induced premalignant gastric lesions [23], particularly in a population-based study.

Based on the above evidence, we performed genotyping analyses for *miR-146a* and *miR-27a* polymorphisms and evaluated their associations with various gastric lesions in 2,380 participants from a gastroscopy-based study in Linqu County, Shandong Province, a high-risk area of GC in China.

## Materials and Methods

### Study Population

From November 1989 through March 1990, a total of 3433 individuals participated in an endoscopic screening survey, representing 83% of eligible residents aged 35–64 years in 14 villages selected at random within four townships of Linqu County, as described previously [24]. The study was approved by the Institutional Review Boards of Peking University Cancer Hospital & Institute, and all participants gave written informed consent. In the current study, a total of 2380 participants providing the blood samples and having gastric histopathologic diagnoses were included. There was no evident difference in basic characteristics between the included and excluded participants (data not shown).

### Histopathology

Details of the gastroscopic procedures and histopathologic criteria have been described elsewhere [3], [24]. Briefly, for each participant, biopsy samples were taken at seven standard sites in the stomach mucosa and given its corresponding histopathologic diagnosis by three senior pathologists independently. Each biopsy was classified according to the presence or absence of superficial gastritis (SG), chronic atrophic gastritis (CAG, mild or severe), IM (superficial or deep), DYS (mild, moderate or severe) or GC. Each biopsy was given a diagnosis based on the most severe histology, and each participant was assigned a global diagnosis based upon the most severe diagnosis among any of the biopsies.

### Blood Sample Collection and DNA Preparation

A 5 ml blood sample was collected from each participant, allowed to clot for 30–40 min at room temperature and then centrifuged at 965 g for 15 min. The resulting serum was separated into vials. The clot and serum were stored immediately at −20°C and then moved into a −70°C freezer at Peking University Cancer Hospital & Institute within 2 or 3 days after collection. High molecular weight genomic DNA was isolated by standard proteinase K digestion and phenol-chloroform extraction from the blood samples.

### *H. pylori* Antibody Assay

As described previously, *H. pylori* antibody assays were used to determine *H. pylori* infection status in 1989 [25]. In brief, serum levels of anti-*H. pylori* IgG and IgA were measured separately in duplicate with enzyme-linked immunosorbent assay procedures. An individual was defined to be positive for *H. pylori* infection if the mean optical density for either IgG or IgA was ≥1.0. Quality control samples were assayed at Vanderbilt University, Nashville, TN.

### Genotyping Analysis

Genotyping was performed by polymerase chain reaction-restriction fragment length polymorphism (PCR-RFLP) analysis. Genomic DNA was amplified in a 20-µl reaction mixture, containing 100 ng of template DNA, 10 pmol of each primer (rs2910164: F: 5′-CATGGGTTGTGTCAGTGTCAGAGCT-3′ and R: 5′-TGCCTTCTGTCTCCAGTCTTCCAA-3′; rs895819: F: 5′-GAACTTAGCCACTGTGAACACCACTTGG-3′ and R: 5′-TTGCTTCCTGTCACAAATCACATTG-3′), 0.0625 mM of each dNTP, 0.5 mM of MgCl_2_ and 0.5 U GoTaq® DNA polymerase in 5× reaction buffer (Promega, Madison, WI). PCR was accomplished by an initial denaturation of 95°C for 2 min, followed by 35 cycles of 95°C for 30 s, 60°C for 45 s and 72°C for 45 s, with a final elongation at 72°C for 5 min.

For RFLP analysis, PCR products were digested with appropriate restriction endonucleases and then visualized using the Ultra Violet gel imaging system on a 3.5% agarose gel that contained 0.5 µg/mL ethidium bromide. For rs2910264 genotyping, a 4.0 µl aliquot of PCR products was digested with 1.0 µl of FastDigest® Sac I in 10× FastDigest® Green Buffer (Fermentas) by incubation at 37°C for 30 min and inactivation at 65°C for 5 min. The genotypes were assessed as follows: a single 147 bp fragment for the GG genotype; two fragments of 122 and 25 bp for the CC genotype; and three fragments of 147, 122, and 25 bp for the GC genotype. For rs895819 genotyping, a 5.0 µl aliquot of PCR products was digested with 0.5 µl of FastDigest® Dra III in 10× FastDigest® Green Buffer (Fermentas, Burlington, Canada) by incubation at 37°C for 5 min and inactivation at 80°C for 5 min. The genotypes were assessed as follows: a single 182 bp fragment for the CC genotype; two fragments of 155 and 27 bp for the TT genotype; and three fragments of 182, 155, and 27 bp for the TC genotype. The genotypes identified by RFLP were further confirmed by DNA sequencing with ABI Prism 377 DNA Sequencer (Applied Biosystems, Foster City, CA).

### Quality Control Procedures

Rigorous quality control procedures were applied throughout genotyping process. To avoid PCR contamination, reagents for PCR were carefully aliquoted and each aliquot was used no more than three times. A negative control (no DNA template) was added in each assay to monitor PCR contamination. A pilot study (50 samples) was conducted to optimize the conditions of PCR and restriction digestion. The electrophoretogram was read by one or two trained technicians blinded to the diagnosis of each participant and independent triplicate experiments were done for dubious samples. After genotyping, approximately 10% to 15% of samples in each genotype group were selected for repeated assays using PCR-DNA sequencing and the concordance rate was >99%.

### Statistical Analysis

Because there were very few participants with normal gastric mucosa or SG in this population, we combined them with mild CAG as one group. Consequently, all participants were divided into four lesion groups: SG/mild CAG (n = 965), severe CAG (n = 204), IM (n = 765) and DYS (n = 446). We used one-way analysis of variance to examine the overall difference in age, and Pearson’s χ^2^ test to test the differences in distributions of categorical variables (i.e. gender, *H. pylori* infection, smoking, drinking and genotype) among groups. We employed a goodness-of-fit χ^2^ analysis to test the Hardy-Weinberg equilibrium.

To assess the associations of genetic variants with gastric lesions, we estimated odds ratios (ORs) and 95% confidence intervals (CIs) using the unconditional multivariate logistic regression model with SG/mild CAG as the reference group, and adjusted for age, gender, *H. pylori* infection, smoking and drinking status. Test for trend was conducted in logistic regression under the codominant model by using a 3-level ordinal variable for each SNP (0 = homozygote wild, 1 = heterozygote, 2 = homozygote variant).

To evaluate whether observed associations varied by *H. pylori* infection, we conducted stratified analyses using unconditional logistic regression. For gene - *H. pylori* infection joint effect analysis, a composite variable with values of 1 to 6 was generated by assembling levels of the two combined factors, and ORs and 95% confidence intervals were estimated by adding this variable to the logistic model after transformation of dummy variables. Potential interactions between polymorphisms and *H. pylori* infection were evaluated on the multiplicative scale by adding a cross-product term between *H. pylori* infection and genotypes of each SNP into the multivariate logistic model. *P* for interaction was calculated using the likelihood ratio test to compare the two models with and without interaction terms (degree of freedom = 2).

All analyses were performed using the Statistical Analysis System software (version 9.1; SAS Institute, Cary, NC). *P* value of <0.05 was considered significant and all statistical tests were two-sided.

## Results

### Participant Information

A total of 2,380 participants (1,180 males and 1,200 females) were enrolled in this study, with the mean age of 45.3±8.2 (Table 1). Information on *H. pylori* infection, smoking, and drinking status was available in 2,078 (87.3%), 2,343 (98.4%) and 2,339 (98.3%) participants, respectively. Significant differences in the overall distributions of age, gender, *H. pylori* infection, smoking and drinking status were identified among four lesion groups. As expected, more elder, male, *H. pylori*-positive, smoking, and drinking persons were found with severe CAG, IM and DYS than with SG/mild CAG.

**Table 1:** Selected characteristics of participants with various gastric lesions.

Variable	SG/mild CAG*n = 965	Severe CAG*n = 204	IM*n = 765	DYS*n = 446	Totaln = 2,380	*P* †
Mean age ± SD*, years	43.5±7.4	43.7±7.7	46.4±8.5	47.8±8.7	45.3±8.2	<0.001
Gender (%)						<0.001
Male	450 (46.6)	97 (47.5)	368 (48.1)	265 (59.4)	1,180 (49.6)	
Female	515 (53.4)	107 (52.5)	397 (51.9)	181 (40.6)	1,200 (50.4)	
*H. pylori* infection (%)						<0.001
Positive	527 (54.6)	159 (77.9)	565 (73.9)	340 (76.2)	1,591 (66.8)	
Negative	284 (29.4)	20 (9.8)	113 (14.8)	70 (15.7)	487 (20.5)	
Missing	154 (16.0)	25 (12.3)	87 (11.3)	36 (8.1)	302 (12.7)	
Smoking (%)						<0.001
Yes	375 (38.9)	81 (39.7)	327 (42.7)	240 (53.8)	1,023 (43.0)	
No	574 (59.5)	123 (60.3)	426 (55.7)	197 (44.2)	1,320 (55.5)	
Missing	16 (1.6)	0 (0.0)	12 (1.6)	9 (2.0)	37 (1.5)	
Drinking (%)						0.001
Yes	420 (43.5)	87 (42.6)	324 (42.4)	236 (52.9)	1,067 (44.8)	
No	528 (54.7)	117 (57.4)	426 (55.6)	201 (45.1)	1,272 (53.4)	
Missing	17 (1.8)	0 (0.0)	15 (2.0)	9 (2.0)	41 (1.8)	

* SG = superficial gastritis; CAG = chronic atrophic gastritis; IM = intestinal metaplasia; DYS = dysplasia; SD = standard deviation.

† *P* value for each covariate was estimated among participants without missing value on that covariate.

### Genotype Distributions

The genotype distributions of the two polymorphisms in various gastric lesion groups are shown in Table 2. The genotype frequencies of these two SNPs in the study population fit the Hardy-Weinberg equilibrium (*P* = 0.08 for rs2910164; *P* = 0.48 for rs895819). Overall, no significant difference was observed in the genotype distributions of the two SNPs among different groups (*P* = 0.08 for rs2910164; *P* = 0.91 for rs895819).

**Table 2:** Genotype distribution and associations of genetic variants with risk of gastric lesions.

Variant	SG/mild CAG*	Severe CAG*		IM*		DYS*	
	n (%)	n (%)	OR (95% CI)* †	n (%)	OR (95% CI)* †	n (%)	OR (95% CI)* †
rs2910164‡							
GG, n (%)	177 (18.4)	30 (14.8)	1.00 (referent)	124 (16.3)	1.00 (referent)	75 (16.8)	1.00 (referent)
GC, n (%)	511 (53.1)	104 (51.2)	1.24 (0.77–1.99)	374 (49.1)	1.14 (0.84–1.54)	212 (47.5)	1.09 (0.76–1.55)
CC, n (%)	275 (28.5)	69 (34.0)	1.40 (0.84–2.34)	264 (34.6)	1.42 (1.03–1.97)	159 (35.7)	1.54 (1.05–2.25)
* P* for trend§			0.21		0.02		0.01
rs895819∥							
TT, n (%)	504 (52.4)	98 (48.3)	1.00 (referent)	402 (53.0)	1.00 (referent)	224 (50.6)	1.00 (referent)
TC, n (%)	389 (40.5)	88 (43.3)	1.33 (0.94–1.88)	300 (39.6)	1.06 (0.84–1.33)	185 (41.8)	1.19 (0.91–1.56)
CC, n (%)	68 (7.1)	17 (8.4)	1.11 (0.59–2.11)	56 (7.4)	0.94 (0.62–1.44)	34 (7.7)	1.05 (0.64–1.73)
* P* for trend§			0.11		0.65		0.20

* SG = superficial gastritis; CAG = chronic atrophic gastritis; IM = intestinal metaplasia; DYS = dysplasia; OR = odds ratio; CI = confidence interval.

† Odds ratios and 95% confidence intervals were estimated in reference to SG/mild CAG group, and adjusted for age, gender, *H. pylori* infection, smoking and drinking status. The analysis was restricted to participants with complete data on all covariates.

‡ Six participants with missing values on rs2910164 genotype were excluded from analysis.

§ *P* for trend were calculated by including the 3-level ordinal variable under codominant model for each polymorphism (0 = homozygote wild, 1 = heterozygote, 2 = homozygote variant) as a continuous variable to the multivariate models.

∥ Fifteen participants with missing values on rs895819 genotype were excluded from analysis.

### Associations of miRNA Polymorphisms with Risk of Gastric Lesions

We firstly evaluated the association between the two SNPs and risk of advanced gastric lesions. As shown in Table 2, participants carrying rs2910164 CC genotype had a significantly increased risk of developing IM (OR, 1.42; 95% CI, 1.03–1.97) and DYS (OR, 1.54; 95% CI, 1.05–2.25) compared to GG genotype, after adjusting for age, gender, *H. pylori* infection, smoking and drinking status. The number of rs2910164 C allele showed a significant dose-response relationship with the risk of IM (*P* for trend_ = _0.02) and DYS (*P* for trend_ = _0.01). No significant association was found for rs895819 and risk of advanced gastric lesions.

We further examined whether *H. pylori* infection modified the associations of SNPs with advanced gastric lesions by stratified analysis. We collapsed the four-category outcome into a binary variable to increase statistical power, and evaluated the association between genetic variants and the risk of IM/DYS in reference to SG/CAG. As shown in Table 3, among participants with positive *H. pylori* infection, those who carried rs2910164 CC genotype had a significantly increased risk of IM/DYS compared to GG carriers (OR, 1.53; 95% CI, 1.12–2.08, *P* for trend = 0.004), whereas such association was not found among participants without *H. pylori* infection (OR, 0.93; 95% CI, 0.52–1.66, *P* for trend = 0.90). For rs895819, no significant association was observed among individuals either with or without *H. pylori* infection.

**Table 3:** Associations of genetic variants with risk of advanced gastric lesions by *H. pylori* infection*.

Variant	*H. pylori* infection	Genotype	SG/CAG†	IM/DYS†		
			n (%)	n (%)	OR (95% CI)† ‡	*P* for trend§
rs2910164∥	Negative	GG	46 (15.2)	32 (17.6)	1.00 (referent)	
		GC	165 (54.5)	91 (50.0)	0.86 (0.50–1.47)	
		CC	92 (30.4)	59 (32.4)	0.93 (0.52–1.66)	0.90
	Positive	GG	124 (18.1)	145 (16.1)	1.00 (referent)	
		GC	359 (52.4)	444 (49.2)	1.15 (0.86–1.53)	
		CC	202 (29.5)	314 (34.8)	1.53 (1.12–2.08)	0.004
rs895819¶	Negative	TT	155 (51.2)	92 (50.6)	1.00 (referent)	
		TC	129 (42.6)	77 (42.3)	1.03 (0.69–1.54)	
		CC	19 (6.3)	13 (7.1)	1.12 (0.50–2.51)	0.79
	Positive	TT	358 (52.5)	472 (52.7)	1.00 (referent)	
		TC	268 (39.3)	357 (39.8)	1.04 (0.84–1.29)	
		CC	56 (8.2)	67 (7.5)	0.92 (0.62–1.36)	0.94

* This analysis was restricted to 2078 participants who had diagnosis for *H. pylori* infection.

† SG = superficial gastritis; CAG = chronic atrophic gastritis; IM = intestinal metaplasia; DYS = dysplasia; OR = odds ratio; CI = confidence interval.

‡ Odds ratios and 95% confidence intervals were estimated in reference to SG/CAG group, and adjusted for age, gender, smoking and drinking status. Participants with missing data on any of covariates were excluded from the analysis.

§ *P* for trend was calculated by including the 3-level ordinal variable under codominant model for each genetic variant (0 = homozygote wild, 1 = heterozygote, 2 = homozygote variant) as a continuous variable to the multivariate models.

∥ Five participants with missing values on rs2910164 genotype were excluded from analysis.

¶ Fifteen participants with missing values on rs895819 genotype were excluded from analysis.

### Gene–*H. pylori* Infection Joint Effect and Interaction

We also explored the joint effects and possible gene–environment interaction between these two SNPs and *H. pylori* infection. As shown in Table 4, compared to participants who were *H. pylori*-negative and carried homogeneous wild genotype of each SNP, participants with *H. pylori* infection alone or carrying both hazard allele and *H. pylori* infection had increased risk of advanced gastric lesions.

**Table 4:** Joint effect and interaction between polymorphisms and *H. pylori* infection on risk of intestinal metaplasia and dysplasia*.

Variant	Genotype	*H. pylori* infection	OR (95% CI)†	*P* for trend‡
rs2910164	GG	Negative	1.00 (referent)	
	GC	Negative	1.01 (0.68–1.50)	
	CC	Negative	1.08 (0.48–2.41)	
	GG	Positive	2.44 (1.80–3.31)	
	GC	Positive	2.55 (1.86–3.49)	
	CC	Positive	2.25 (1.43–3.54)	<0.001
	*P* for interaction§		0.35	
rs895819	TT	Negative	1.00 (referent)	
	TC	Negative	0.87 (0.51–1.48)	
	CC	Negative	0.93 (0.52–1.66)	
	TT	Positive	1.83 (1.09–3.10)	
	TC	Positive	2.09 (1.28–3.39)	
	CC	Positive	2.77 (1.68–4.56)	<0.001
	*P* for interaction§		0.92	

* This analysis was restricted to 2078 participants who had diagnosis for *H. pylori* infection.

† Odds ratios (ORs) and 95% confidence intervals (CI) for the risk of intestinal metaplasia and dysplasia were estimated in reference to superficial gastritis/chronic atrophic gastritis, and adjusted for age, gender, smoking and drinking status. Participants with missing data on any of variables were excluded from the analysis.

‡ *P* for trend was calculated by assigning an ordinal value (1–6) to each combination of genotype and *H. pylori* infection (1 = homozygote wild without *H. pylori* infection, 2 = heterozygote without *H. pylori* infection, 3 = homozygote variant without *H. pylori* infection, 4 = homozygote wild with *H. pylori* infection, 5 = heterozygote with *H. pylori* infection, 6 = homozygote variant with *H. pylori* infection) and adding it as a continuous variable to the multivariate model.

§ Likelihood ratio test was used to calculate the *P* value for interaction by comparing the two models with and without the product term of genotypes and *H. pylori* infection.

For rs2910164, individuals who simultaneously carried CC genotype and had *H. pylori* infection were at a 2.25-fold increased risk of developing IM/DYS compared to those carrying GG genotype and without *H. pylori* infection (95% CI, 1.43–3.54, *P* for trend <0.001). Similarly, for rs895819, the coexistence of CC genotype and *H. pylori* infection was associated with a higher risk of IM/DYS (OR, 2.77; 95% CI, 1.68–4.56, *P* for trend <0.001). However, the formal test for gene–*H. pylori* infection interaction did not attain statistical significance for either rs2910164 or rs895819 (*P* for interaction = 0.35 and 0.92, respectively).

## Discussion

In the present study, based on our previous miRNA array data, we selected two potential functional SNPs (rs2910164 in *miR-146a* and rs895819 in *miR-27a*) and investigated their relationship with *H. pylori*-associated gastric lesions in a Chinese population. We found that rs2910164 may affect the susceptibility of gastric lesions. To our best knowledge, this is the first study to evaluate the associations between miRNA polymorphisms and precancerous gastric lesions in a large population. Our findings suggest that genetic variants of miRNA may play a role in *H. pylori*-related gastric pathogenesis.

The rs2910164 G>C polymorphism is located in the stem region opposite to the mature *miR-146a* sequence and results in a change from G:U pair to C:U mismatch in the stem structure of *miR-146a* precursor [11]. Several studies have reported the associations between rs2910164 and multiple malignancies, including prostate, hepatocellular, papillary thyroid and esophageal squamous cell carcinoma [11], [21], [26], [27]. As for GC, the results have been mixed. A Chinese study without adjusting for *H. pylori* infection showed that persons carrying rs2910164 GG/GC genotype were at significantly increased risk of GC compared to CC genotype [14], while a Japanese study demonstrated that individuals carrying C allele were more likely to develop gastric carcinoma than GG carriers after adjusting for *H. pylori* infection [22]. However, a study in the Korean population did not observe a significant association between rs2910164 and risk of GC [28]. A recent meta-analysis found that rs2910164 GG/GC genotype was significantly associated with increased overall cancer susceptibility in Asians [29]. Our results are consistent with the Japanese report, suggesting that rs2910164 CC carriers had a higher risk of IM and DYS after controlling for potential confounders including *H. pylori* infection. We also found that the association between rs2910164 CC genotype and risk of IM and DYS appeared to be more pronounced in *H. pylori*-positive individuals.

*MiR-146a* has been implicated in control of toll-like receptors (TLRs) and cytokine signaling pathway, reducing NF-κB activity through negatively regulating IRAK1 and TRAF6 expressions [16], [30]. It’s well known that TLR2, 4, 5 and 9 are involved in *H. pylori* recognition [31] and NF-κB is a key molecule in inflammation-cancer link [32]. Several studies provided the experimental evidences that rs2910164 C allele in *miR-146* precursor could reduce mature miR-146a production [11], [21], which might modify the inflammatory process. Although the exact mechanism of *miR-146a* polymorphism on the *H. pylori*-associated pathogenesis is unclear, it is possible that rs2910164 G>C polymorphism might interfere the normal immune response to *H. pylori* infection and thus contribute to the elevated risk of advanced gastric lesions, such as IM and DYS. The functional relevance of this polymorphism as well as the exact mechanism in the *H. pylori*-associated carcinogenesis needs to be confirmed.

The rs895819 T>C polymorphism is located in the *miR-27a* precursor, at position 40 relative to the first nucleotide. *MiR-27a* is located at chromosome 19 and has been shown to function as oncogene by targeting *prohibitin* [13], *FOXO1* [17] and *Sprouty2* [33] in various cancers including GC. Accumulating evidence from basic research suggests that prohibitin and FOXO1 may play important roles in inflammatory process by modulating early inflammatory responses [34] and protecting inflammation-associated oxidative injury [18], [35], [36]. However, epidemiologic evidence on the association between rs895819 polymorphism and GC and precancerous gastric lesions is sparse [20], [23], [37].

Recently, a Chinese study found that rs895819 TC/CC genotype was associated with an increased risk of GC by enhancing miR-27a production and reducing the mRNA level of its target gene *ZBTB10* [20]. However, this association was not replicated by another study [37]. A Japanese study examined the relationship between rs895819 and chronic gastritis in 179 participants without gastric malignancy, and observed that CC carriers had significantly higher atrophy and metaplasia scores than TC/TT carriers in men [23]. In the current study, we did not observe a significant association between rs895819 and risk of gastric lesions. Given the sparse literature [38], however, further studies are needed to investigate the relationship of rs895819 with gastric carcinogenesis.

We were also interested to evaluate the possible joint effect and interaction between rs2910164 G>C or rs895819 T>C polymorphism and *H. pylori* infection. A significantly increased risk of IM and DYS was observed in participants with *H. pylori* infection alone, consistent with our previous finding in Linqu that *H. pylori* infection played an important role in the incidence and progression of gastric lesions [25]. Further analysis revealed a significant joint effect between rs2910164, rs895819 and *H. pylori* infection, suggesting a gene-environmental interplay may modify *H. pylori*-related carcinogenesis and outcome of *H. pylori* infection. However, given the limited sample size, the formal tests failed to detect any significant interaction. Since successful identification of gene–*H. pylori* interaction could help discern the intricate mechanism of gastric carcinogenesis and explain the high variation in the GC incidence observed both around the world and within the high *H. pylori* prevalence population, more and larger population studies are deserved in this area [39].

Our study has both strengths and limitations. The strengths of this study include the relatively large homogeneous population, well-defined histopathological diagnosis, and detailed information on potential confounders, especially *H. pylori* infection. In addition, we selected the potential functional SNP loci from the differentially expressed miRNAs based on the serum profiling of GC. This approach increased the possibility that our identified SNP loci were biologically relevant to gastric carcinogenesis.

One limitation of this study was the combination of SG/mild CAG as controls in overall analysis because very few participants were diagnosed with normal gastric mucosa in this population [24]. However, this “sub-normal” control could only lead to the dilution of disparity between comparison groups and in turn attenuate the magnitude of association. In addition, we converted the outcome from four categories into a binary variable (IM/DYS vs. SG/CAG) to preserve statistical power in stratification and interaction analysis, which may lead to outcome misclassification and loss of information. Another potential drawback is that we did not analyze the SNPs in the target genes of these miRNAs. Since miRNAs indirectly play biological roles through regulating target mRNA expression, SNPs in both miRNA and its target genes may interactively affect the progression of precancerous gastric lesions.

In summary, our population-based study provided evidence that rs2910164 polymorphism in *miR-146* was associated with advanced gastric lesions. Rs2910164 polymorphism might promote the occurrence of intestinal metaplasia and dysplasia jointly with *H. pylori* infection. Further functional validation of these observational findings needs to be conducted.
