# Correction: Genetic Polymorphisms of *miR-146a* and *miR-27a*, *H. pylori* Infection, and Risk of Gastric Lesions in a Chinese Population

**DOI:** 10.1371/annotation/7c922a5d-8998-45c3-a15b-6dbbe54aac42

**Published:** 2014-01-22

**Authors:** Ming-yang Song, Hui-juan Su, Lian Zhang, Jun-ling Ma, Ji-you Li, Kai-feng Pan, Wei-cheng You

The first and second authors, Ming-Yang Song and Hui-juan Su, should be noted as having contributed equally to the article.

